# Enhancing the sensitivity of a surface plasmon resonance-based optical sensor for zinc ion detection by the modification of a gold thin film

**DOI:** 10.1039/c9ra07368j

**Published:** 2019-12-17

**Authors:** Wan Mohd Ebtisyam Mustaqim Mohd Daniyal, Yap Wing Fen, Nur Ain Asyiqin Anas, Nur Alia Sheh Omar, Nur Syahira Md Ramdzan, Hideki Nakajima, Mohd Adzir Mahdi

**Affiliations:** Institute of Advanced Technology, Universiti Putra Malaysia 43400 UPM Serdang Selangor Malaysia yapwingfen@upm.edu.my; Department of Physics, Faculty of Science, Universiti Putra Malaysia 43400 UPM Serdang Selangor Malaysia; Synchrotron Light Research Institute Maung Nakhon Ratchasima 30000 Thailand; Wireless and Photonics Networks, Faculty of Engineering, Universiti Putra Malaysia 43400 UPM Serdang Selangor Malaysia

## Abstract

Surface plasmon resonance (SPR) sensors as novel optical sensors for the detection of a variety of analytes have been receiving increasing attention and their sensitivity has become the research hotspot recently. In this study, the sensitivity of an SPR optical sensor was enhanced by modifying a gold thin film with a nanocrystalline cellulose (NCC)-based material for zinc ion (Zn^2+^) detection that exists in the environment due to industrial processing. By replacing the gold thin film with a novel modified-gold thin film, Zn^2+^ can be detected from the range of 0 to 10 ppm using SPR. It is believed that the Zn^2+^ may interact with the negative charge molecules that exist on the modified-gold thin film, and this was confirmed *via* X-ray photoelectron spectroscopy (XPS). Moreover, this modified-gold-SPR has a high sensitivity of 1.892° ppm^−1^ up to 0.1 ppm with an enhanced detection of Zn^2+^ as low as 0.01 ppm. The SPR results also followed the Langmuir isotherm model with a binding affinity of 1.927 × 10^3^ M^−1^, which further confirmed the sensitivity of the SPR sensor. In addition, using the modified-gold thin film, SPR has a higher affinity towards Zn^2+^ compared to other metal ions, *i.e.* Ni^2+^, Fe^2+^, Cr^2+^, Mn^2+^, and Co^2+^.

## Abstract 2

This work focus on sensitivity enhancement of surface plasmon resonance (SPR) optical sensor by modifying the gold thin film with nanocrystalline cellulose (NCC) based material for zinc ion (Zn^2+^) detection.

## Graphical abstract

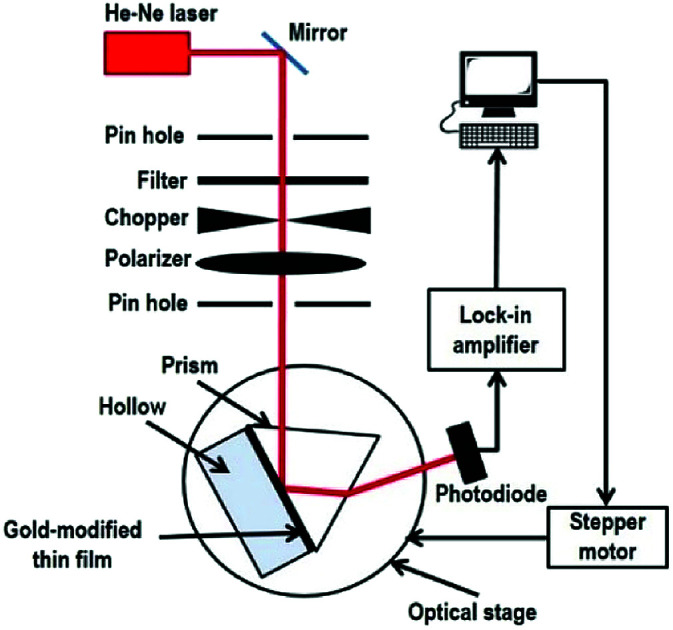

## 1. Introduction

A biosensor or sensor is an analytical tool that is used for the determination of analytes based on a biocatalyst type of sensors.^1^ A sensor can be dependent on magnetic, mechanical, electrical, and optical principles. Among all, optical sensors are the most simpler in their setup for data acquisition and working activity. An optical sensor has many advantages over electrical and mechanical sensors, such as the optical sensor does not modify nor destruct the measured and/or the surrounding environment.^2^ Basically, optical sensors require a recognition element that can interact specifically with the desired target analytes and then, the sensor will detect the signal of the binding event.^3^ For instance, reflection interference spectroscopy (RIFS),^4^ surface-enhanced Raman scattering (SERS) spectroscopy,^5^ and surface plasmon resonance (SPR) are optical sensors that require a recognition element.^6^ SPR is one of the favorable optical sensors that is widely used in sensing biochemical reaction owing to its advantages such as low-cost, label-free, fast measurement, and simple sample preparation.^7^

The most common setup for SPR is by the Kretschmann configuration. When a p-polarized light is incident onto a metallic thin film (typically gold or silver) through the prism, the free electrons on the metal surface will excite and form a surface plasmon. At a certain angle, the surface plasmon will then resonate with the incident light, thus reducing the intensity of the reflected light.^8^ This angle is known as the resonance angle. SPR is very sensitive towards the changes in the refractive index of the metal surface. Such change may result in a change in the resonance angle.^9^ However, SPR sensitivity is limited, where any solution with the same refractive index, such as a low concentration of the metal ion solution.^10^ Sensitivity is one of the most important features of a sensor where it is affected by the bioreceptor, biomolecule immobilization procedure or transduction method.^11^ Since the past two decades, SPR has been extensively studied to enhance the optical sensor sensitivity.

One of the strategies to enhance the SPR sensitivity for the metal ion detection is by combining the SPR with other sensing methods. For instance, Wang *et al.* in 2007 combined the SPR sensor with anodic stripping voltammetry (ASV) for sensing copper, lead, and mercury ions, while Panta *et al.* in 2009 combined the SPR with ASV and magnetohydrodynamic (MHD) convection for sensing mercury ions.^12,13^ Instead of combining SPR with a different technique, researchers also studied the sensitivity of SPR by using different light sources. Eum *et al.* in 2003 modified an SPR sensor with near-infrared (NIR) light sources and they reported that the modified SPR system was able to detect potassium ions.^14^ In 2008, Chen *et al.* used white light sources for the SPR sensor where the detection of uranyl ions is based on a wavelength shift instead of a resonance angle.^15^

Although the SPR sensitivity was proven to be enhanced either by combining the SPR with other methods or by modifying the SPR sensor, the simplest way for the sensitivity enhancement is by modifying a gold thin film with a sensing element. As the SPR sensor works by measuring the refractive index changes in the vicinity of the gold thin film in response to biomolecular interactions, the modification of the gold thin film surface will definitely enhance the SPR sensitivity. Studies on the SPR sensitivity enhancement by the modification of the metal thin film for metal ion detection also has been conducted since 2001.^16–34^ However, only a few studies for sensing zinc ions has been conducted. For example, Wu and Lin in 2004 immobilized metallothionein onto a carboxymethylated dextran matrix to be incorporated with SPR.^35^ They reported that the SPR system can be used to detect zinc as low as 0.13 ppm. In 2011, Fen *et al.* introduced a chitosan layer on top of a gold thin film using glutaraldehyde as the crosslinking agent.^36^ From their report, the SPR sensitivity for zinc ion detection was enhanced as low as 0.5 ppm. In another interesting study, Sadrolhosseini *et al.*, in 2013, used a polypyrrole–chitosan layer for zinc ion detection using SPR.^37^ The binding interactions of the zinc ions with the active layer was monitored using the resonance angle shift where the lowest detection was 0.98 ppm. Fen *et al.* in 2015 again used chitosan and chitosan–tetrabutylthiuram disulphide for the detection of zinc ions using SPR.^38^ The active layer was deposited on the top of the gold layer *via* a spin coating technique before combining with the SPR system to monitor the zinc ion concentration. They found that the SPR system was able to detect zinc ion as low as 0.1 ppm. Although SPR has been studied to detect zinc ion since 2004, the lowest concentration detected was 0.1 ppm. Hence in this study, we modified the gold thin film with a nanocrystalline cellulose (NCC)-based composite that is believed to enhance the sensitivity of the SPR optical sensor for sensing zinc ions at a lower concentration owing to its toxic effect to human.

After iron, zinc is the second most abundant metal ion that exists in the human body, which is about 2–3 g in total.^39^ Zinc is also one of the essential trace elements in biological systems as it is involved in numerous aspects of cellular metabolism, plays a role in the human immune systems and development during pregnancy, supports normal growth, and it is required for a normal sense of smell and taste.^40,41^ Moreover, zinc is estimated to bind about 10% of human proteins, and it is required for the catalytic activity for more than 200 enzymes.^42,43^ Zinc deficiency in the human body can cause damage to the immune function, delay growth, and cause a loss in appetite.^44,45^ Although zinc is very crucial in human biological systems, an excess amount of zinc also proves to be lethal.^46^ At a higher level, zinc can cause health problems such as vomiting, nausea, stomach ache, anemia, and skin problems.^47^ The high level of zinc concentration in the environment is due to industrial sources, such as toxic waste sites, steel processing, waste combustion, and mining. Furthermore, the zinc concentration magnified in tap water is due to the leaching of zinc from fitting and piping.^48^ Therefore, the detection of zinc at a low level is important for the continuous monitoring of environmental water.

## 2. Experimental details

### 2.1. Modified-gold thin film

All chemicals were purchased from Sigma Aldrich (St. Louis, MO, USA). In order to modify the gold thin film with the NCC-based material, hexadecyltrimethylammonium bromide (CTA) was used for a slight modification of NCC. This modification was necessary in order to alter the NCC hydrophilicity properties to make slightly hydrophobic.^49^ NCC was modified by diluting 100 ml of NCC (0.1 wt% suspension) and mixed with 0.1 wt% CTA. The mixture was then centrifuged for 10 minutes before being dispersed with graphene oxide (GO) in a 1 : 1 volume ratio.

### 2.2. Surface plasmon resonance system

**Fig. 1:**
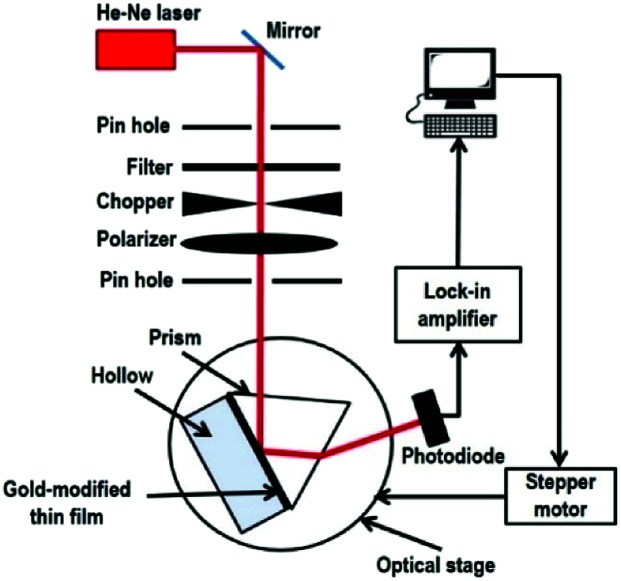
SPR optical sensor setup.

Our custom build SPR optical sensor system is based on Kretschmann configuration that consists of a He–Ne laser beam (632.8 nm, 5 mW), optical chopper (SR 540), polarizer, stepper motor (Newport MM 3000), prism (*n* = 1.77861), photodiode detector, and lock-in amplifier (SR 530), as shown in Fig. 1. In order to generate SPR, the incident light must be in a transverse magnetic mode as the electric field is perpendicular to the metal thin film that can be described by:1
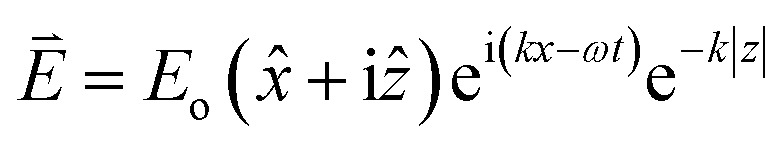
where *E*_o_ is the amplitude, *k* is the wave vector, *x̂* and *ẑ* are the unit vectors, and *ω* is the angular optical frequency of the electrical field. When total internal reflection occurred, surface plasmon was generated by the evanescent wave at the metal and dielectric interface. The surface plasmon wave vector, *K*_sp_ is described by the following equation:2
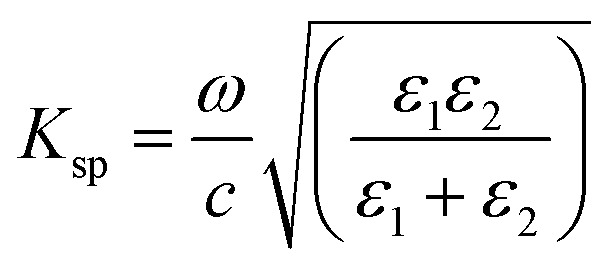
where *ω* is the frequency, *c* is the light velocity, and *ε*_1_ and *ε*_2_ are the dielectric constants for the surface-active and dielectric media, respectively. The dielectric constant can be described by:

3 *ε* = *n*^2^

thus eqn (2) can be rewritten as:4
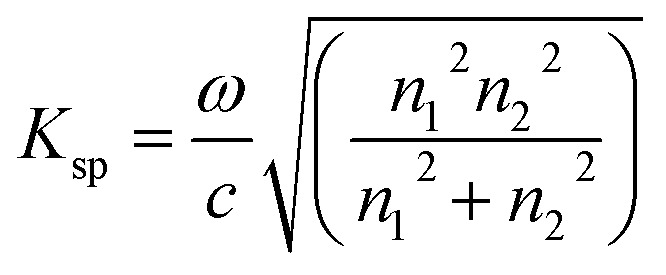
where *n*_1_, *n*_2_, and *n*_p_ are the refractive index of the gold layer, sample, and prism, respectively. SPR occurs when a component of the incident light vector parallel to the prism/metal interface, *K*_x_, given by:5
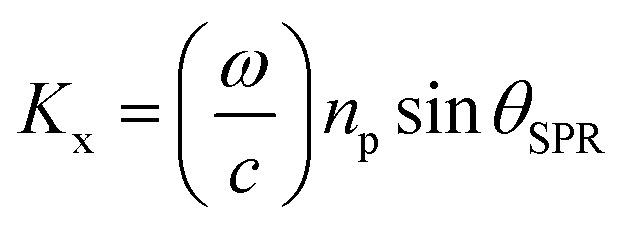
was similar to the surface plasmon wave vector:

6 *K*_sp_ = *K*_x_

with7
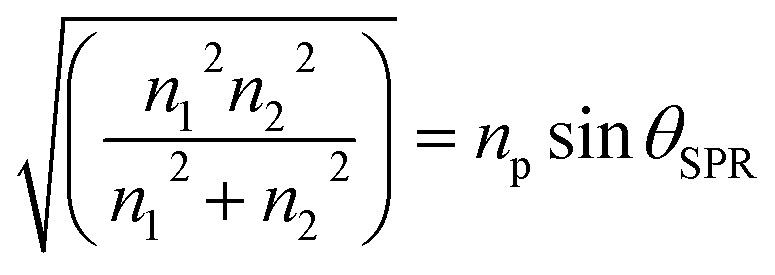


The coupling of these two-wave vectors, *K*_sp_, and *K*_x_ result in a sharp dip of the reflectance at a resonance angle, *θ*_SPR_. The SPR optical sensor works by detecting the changes in the thin film surface refractive index. Thus, the refractive index of the sample is:8
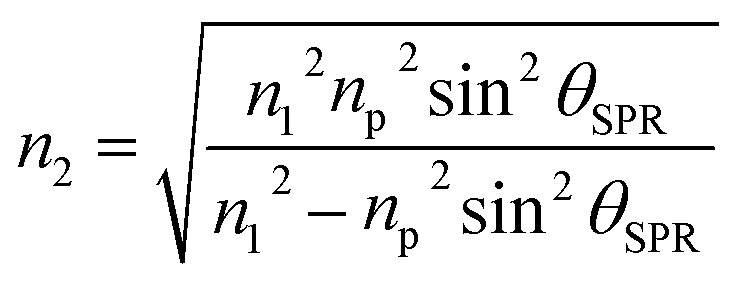


In accordance with the boundary conditions for the electrical and magnetic fields at the interfaces between multilayers, the reflection coefficient, *r*, can be expressed as:9
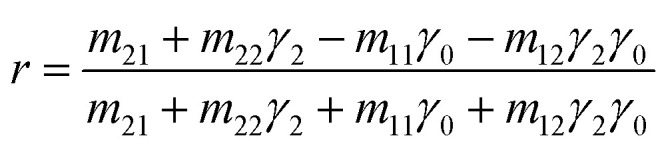
where *m*_*ij*_ is the matrix transfer element. The transfer matrix can be obtained from the relation between the electrical and magnetic layers in the first and the last layers.^50^ The reflectivity of the multilayer system, *R*, is defined as the ratio of the energy reflected at the surface to the energy of the incident, and can be expressed as:

10 *R* = rr*

where it is the function of the refractive index of the sample, the refractive index and thickness of both gold and sensor layer.

To begin with, the SPR experiment was divided into two parts. The first part was to investigate the SPR signal using an unmodified gold thin film to obtain the properties of the resonance angle. Then, the second part of the SPR experiment was performed using the modified-gold thin film to determine the sensing ability. The SPR signal was investigated using both of the thin films in contact with the deionized water and Zn^2+^ of various concentrations, from 0.01 ppm to 10 ppm (prepared by diluting 1000 ppm standard zinc solution with deionized water using the dilution formula *M*_1_*V*_1_ = *M*_2_*V*_2_) injected into the hollow one by one. The SPR curve was taken after Zn^2+^ was injected and left for 10 minutes in the cell.^7^ The SPR signal results for both the modified and unmodified gold thin film contact with deionized water and Zn^2+^ have been discussed in the next section.

### 2.3. X-ray photoelectron spectroscopy

X-ray photoelectron spectroscopy (XPS) was performed to investigate the possible interactions between the modified-gold thin film with Zn^2+^. The XPS study was performed using PHI5000 Versa Probe II, ULVAC-PHI Japan at the SUT-NANOTEC-SLRI Joint Research Facility, Synchrotron Light Research Institute (SLRI), Thailand. The XPS scans were recorded in the range of 0 to 1400 eV and fitted by the Gaussian–Lorentzian curve fitting program with a linear background for each peak in order to determine the binding energies of various element core levels.

## 3. Results and discussion

### 3.1. SPR sensor using an unmodified gold thin film

**Fig. 2:**
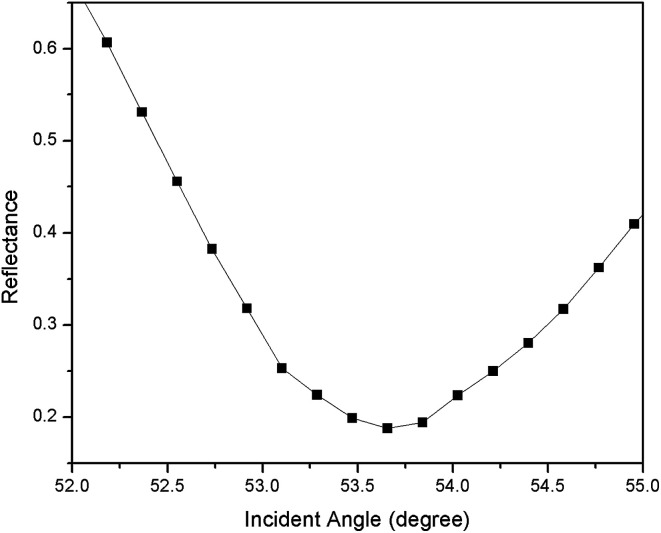
The SPR signal of deionized water using an unmodified gold thin film.

**Fig. 3:**
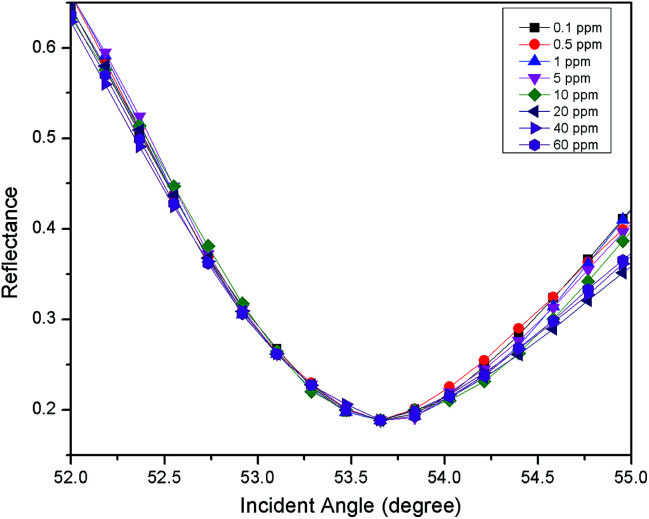
The SPR signal of Zn^2+^ (0.01–10 ppm) using an unmodified gold thin film.

For the first part of the SPR experiment, the resonance angle for deionized water in contact with an unmodified gold thin film obtained was 53.66°, as shown in Fig. 2. The experiment then continued using Zn^2+^ solution. As shown in Fig. 3, the resonance angle of Zn^2+^ for all concentration remain the same as the resonance angle of deionized water, *i.e.* 53.66°. This result might be due to a small binding interaction amount of Zn^2+^ with the gold surface, thus does not change the optical properties of the thin film.^51^ Moreover, the refractive index of metal ions at any concentration below 100 ppm is almost equal to the refractive index of deionized water.^10^

### 3.2. SPR sensor using a modified-gold thin film

**Fig. 4:**
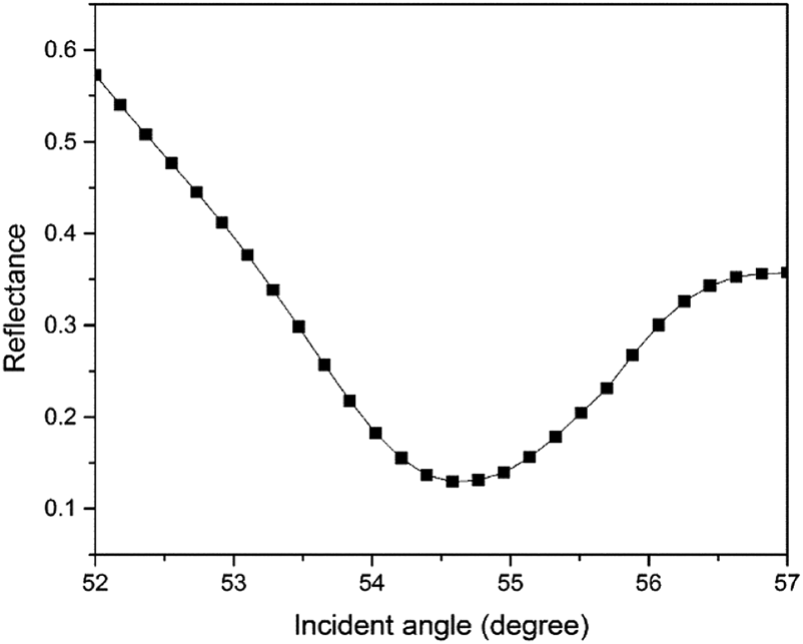
SPR signal of deionized water using the modified-gold thin film.

**Fig. 5:**
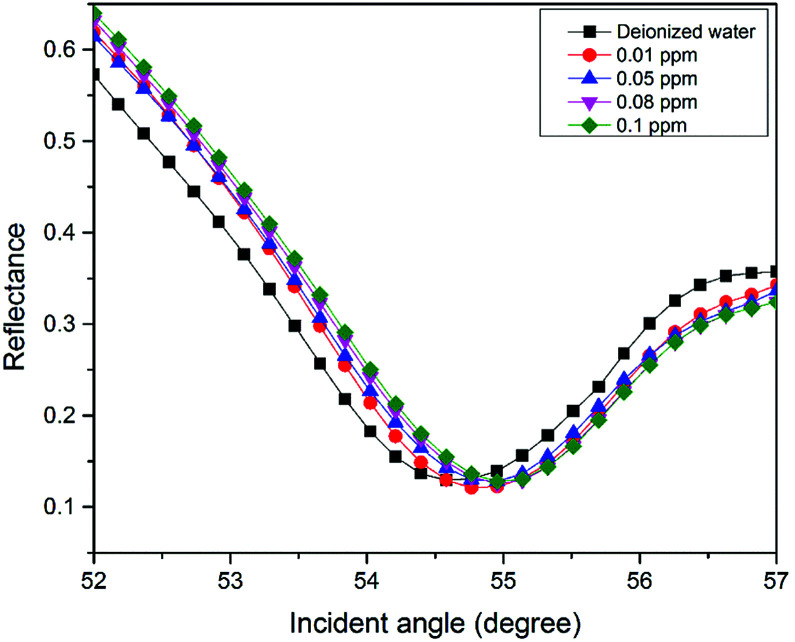
SPR signal of Zn^2+^ solution (0–0.1 ppm) using the modified-gold thin film.

**Fig. 6:**
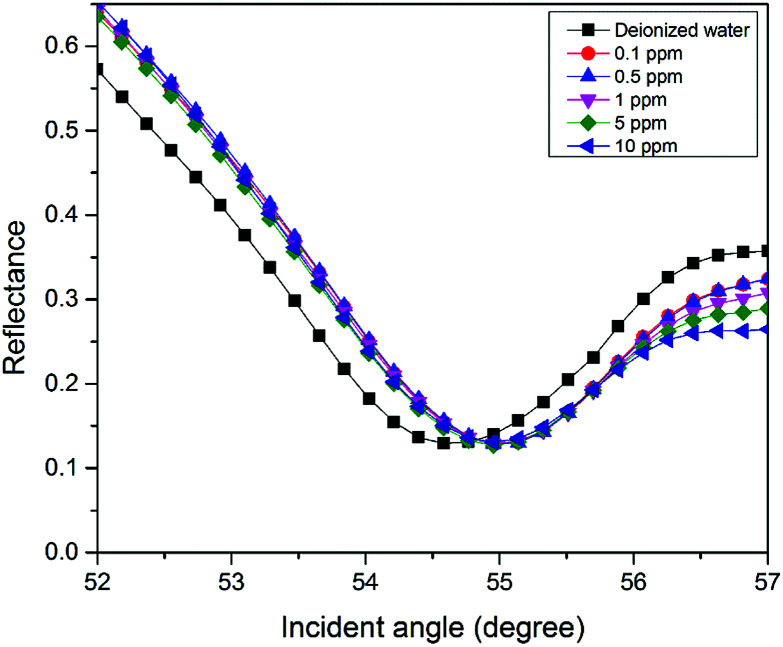
SPR signal of Zn^2+^ solution (0.1–10 ppm) using the modified-gold thin film.

For the second part of the SPR experiment, the resonance angle for deionized water using a modified-gold thin film found was 54.65°, slightly different when using the unmodified gold thin film, as shown in Fig. 4. The unequal resonance angle of deionized water might be due to the refractive index changes when the gold thin film was immobilized with the NCC-based material.^52^ The SPR curves using the modified-gold thin film for sensing Zn^2+^ are shown in Fig. 5 and 6. The SPR curve shifted slightly from the deionized water at 0.01 ppm and shifted further at higher concentrations. The modified-gold thin film achieved saturation after the binding of Zn^2+^ that minimized the changes in the surface refractive index.^53^ Hence, the resonance angle of the SPR curves remains almost at the same at 0.01 until 10 ppm and it can be concluded that the SPR using the modified-gold thin film can be used to detect Zn^2+^ from 0.01 up to 0.1 ppm. Zn^2+^ may bind with the negative charge that exists on the thin film surface forming a pair of shared electrons between the positive charge Zn^2+^, thus changing the optical properties of the thin film.^54^ In order to investigate the existence of the negative charge functional group, the modified-gold thin film was characterized by XPS.

### 3.3. XPS analysis on the modified-gold thin film

**Fig. 7:**
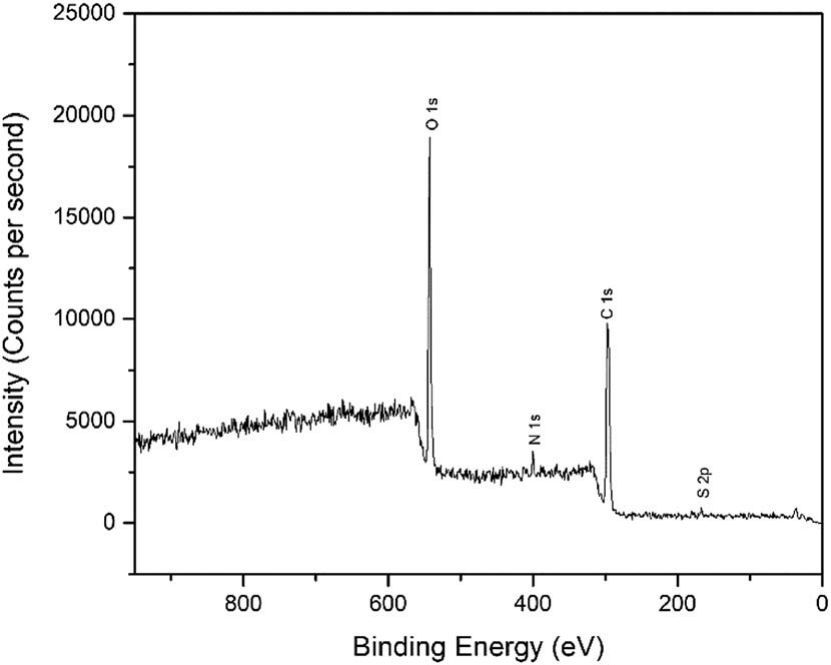
Wide-scan XPS spectra for chemical compositions of the modified-gold thin film.

**Fig. 8:**
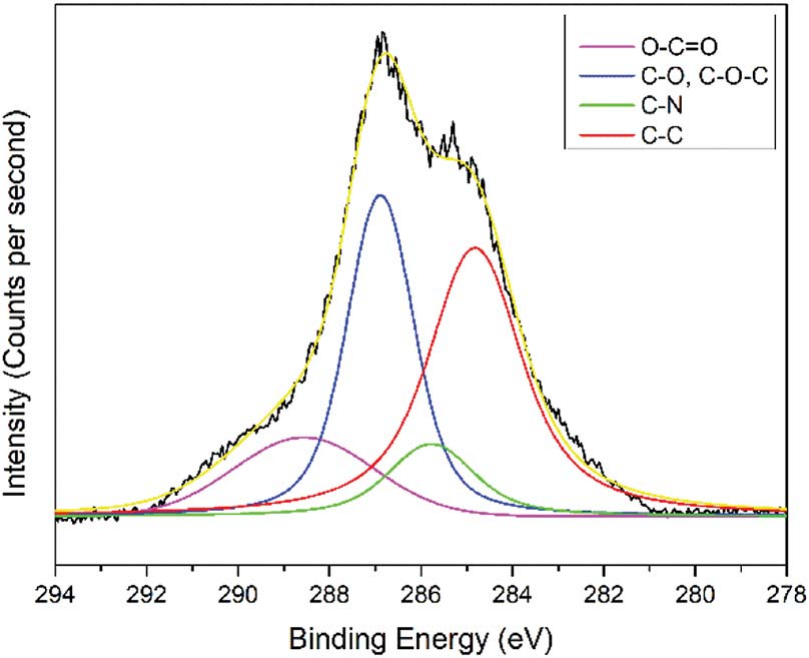
Narrow-scan of the C 1s spectra for the modified-gold thin film.

**Fig. 9:**
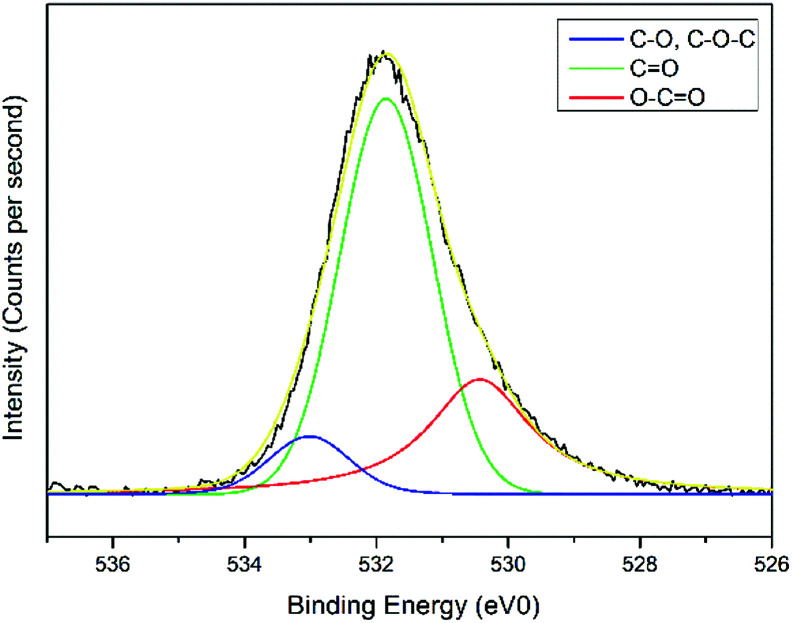
Narrow-scan of O 1s spectra for the modified-gold thin film.

**Fig. 10:**
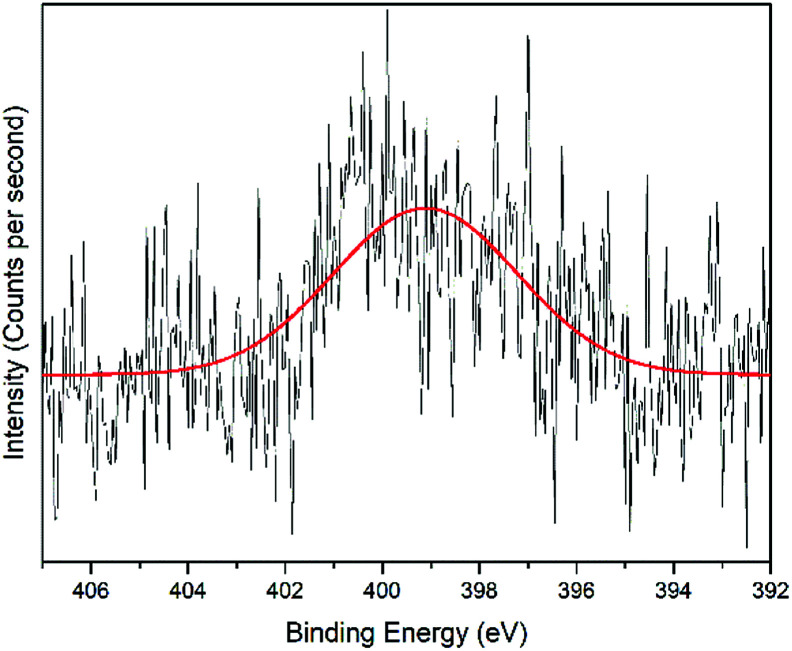
Narrow-scan of N 1s spectra for the modified-gold thin film.

**Fig. 11:**
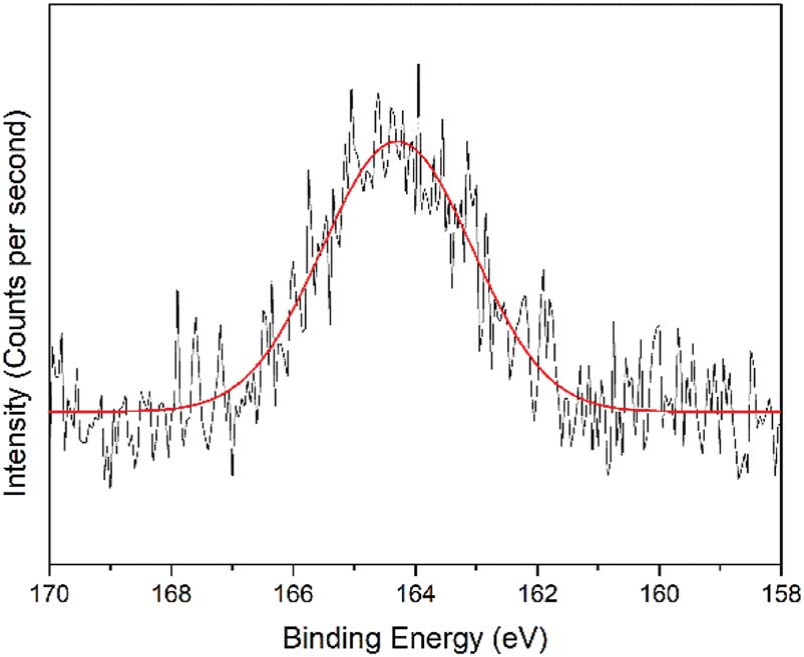
Narrow-scan of S 2p spectra for the modified-gold thin film.

The interactions of NCC, CTA, GO, and gold to form the modified-gold thin film was investigated using XPS. Wide-scan spectra detailing the elemental analysis for the modified-gold thin film is shown in Fig. 7. The studied thin film consists of C 1s, O 1s, N 1s, and S 2p, as confirmed by the spectra. The narrow scan for C 1s, O 1s, N 1s, and S 2p are shown in Fig. 8–11, respectively.

The carbon signal in Fig. 8 can be resolved into several component peaks, which reflect the local environments of the carbon atoms. The C 1s peak at 284.8 eV can be assigned to the C–C chemical binding. The peak at 285.8 eV was assigned to the C–N quaternary ammonium groups due to the modification of NCC.^55^ The peak at 286.9 eV was assigned to the C–O or C–O–C bond. The last peak at 288.9 eV was assigned to the O–C=O or C=O bond. For the O 1s spectrum, the result of the fit assumption is shown in Fig. 9 where three peaks were identified. The peak at 530.4 eV was assigned to O–C=O, the peak at 531.8 eV was assigned to the C=O chemical binding. The last peak at 533.0 eV was assigned to the C–O or the C–O–C chemical binding. Moreover, the spectrum for N 1s was deconvoluted into quaternary-N at 401.0 eV, while for the S 2p spectrum, it was assigned to the sulfonate functional group at 167.4 eV, as shown in Fig. 10 and 11, respectively.^56,57^

From the XPS results, it is believed that the Zn^2+^ may have interacted with the COO^−^ or SO_3_^−^ functional groups that may exist on the modified-gold thin film surface. It was also suggested that COO^−^ played a more important role during the interactions with Zn^2+^ compared to SO_3_^−^ owing to its higher amount on the modified-gold thin film surface. The presence of these negative charge functional groups promotes the electrostatic interactions between Zn^2+^ and the modified gold thin film, thus altering the optical properties of the thin film that causes the shift in the resonance angle.^58^ These results proved that the modification of a gold thin film helps to enhance the sensitivity of SPR.

### 3.4. Sensitivity and binding affinity of SPR using the modified-gold thin film

The sensitivity of the SPR-based optical sensor using the gold-modified thin film was determined by deriving the gradient of the resonance angle shift against the Zn^2+^ concentrations graph from 0.01 until 10 ppm. Overall, it could be seen that the sensitivity of SPR was enhanced by using the modified-goldthin film. The sensitivity of the SPR using the modified-gold thin film toward Zn^2+^ showed high sensitivity from 0.01 until 0.1 ppm, while at a higher concentration the sensitivity decreased. For further analysis, the data was plotted differently to obtain the best linear regression coefficient *R*^2^.

**Fig. 12:**
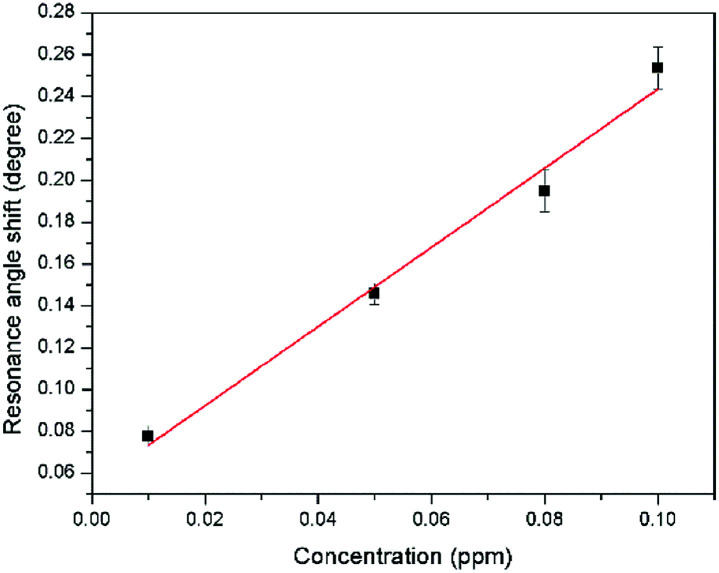
Comparison of the resonance angle shift for the modified-gold thin film with Zn^2+^ concentration (0.01–0.1 ppm).

**Fig. 13:**
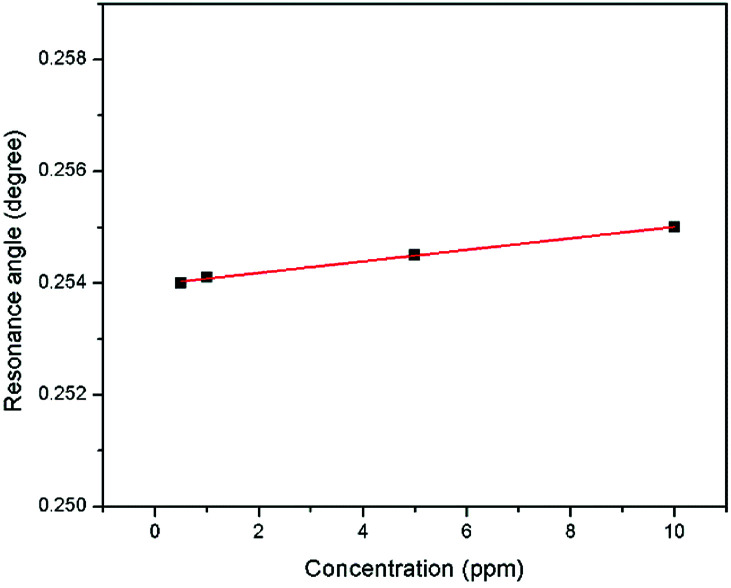
Comparison of the resonance angle shift for the modified-goldthin film with Zn^2+^ concentration (0.5–10 ppm).

The plot to calculate the SPR sensitivity using the modified-gold thin film from 0.01 until 0.1 ppm is shown in Fig. 12, and it can be observed that the resonance angle shift increased linearly with the Zn^2+^ concentration. The linear regression analysis of the graph produces the gradient of 1.892° ppm^−1^, which also represents the sensor sensitivity with an *R*^2^ of 0.96. The SPR sensitivity may reach a saturated value at 0.5 until 10 ppm as the sensitivity of the SPR decreases down to 0.000131° ppm^−1^ with an *R*^2^ of 0.99, as shown in Fig. 13. Moreover, the efficiency of this SPR sensor was compared with other reported studies on the Zn^2+^ ion detection using the SPR method, as summarized in Table 1. The NCC-based material that was used to modify the gold thin film in this study has been proven to detect Zn^2+^ as low as 0.01 ppm, which was much lower as compared to previously reported study, which was at 0.1 ppm. This result may be due to the existence of different negative charge functional groups that exist on the NCC-based composite, *i.e.*, COO^−^ and SO_3_^−^. The COO^−^ and SO_3_^−^ may have had higher electronegativity to attract Zn^2+^ for electrostatic interactions, and hence enhance the SPR sensitivity as compared to the chitosan–tetrabutylthiuram disulfide that was reported to have only sulfur donor atoms.

**Table 1:** Comparison of the lowest detection of Zn^2+^ with various modification of the gold thin film

Sensing layer	Lowest detection	Reference
Polypyrrole–chitosan	0.98 ppm	37
MMW chitosan (glutaraldehyde-crosslinked)	0.5 ppm	36
Immobilize metallothionein onto a carboxymethylated dextran matrix	0.13 ppm	35
Chitosan and chitosan–tetrabutylthiuram disulfide	0.1 ppm	38
Modified-nanocrystalline cellulose/graphene oxide	0.01 ppm	This study

**Fig. 14:**
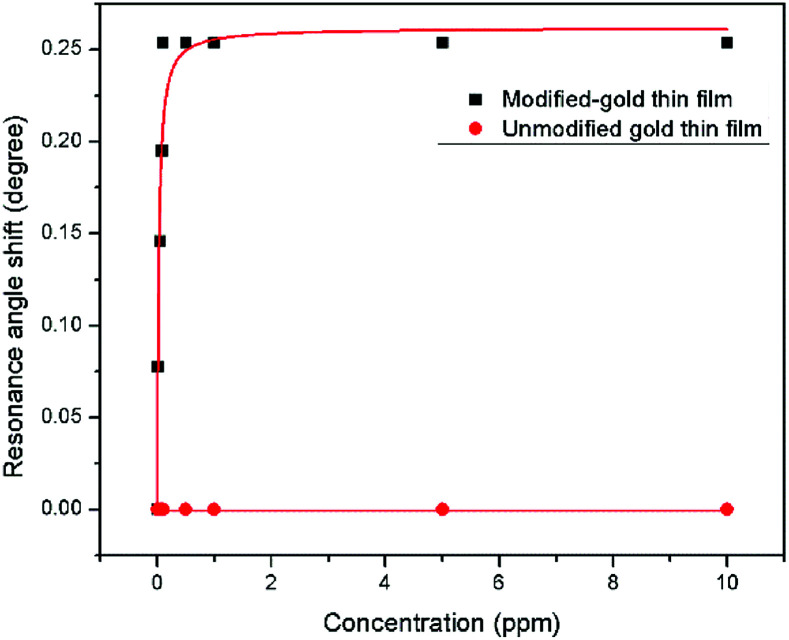
Langmuir isotherm model of the resonance angle shift for modified and unmodified gold thin films in contact with Zn^2+^ ions.

Another important parameter of a sensor is the binding affinity. The binding affinity can be calculated by the Langmuir isotherm model with the following equation.^59–61^11
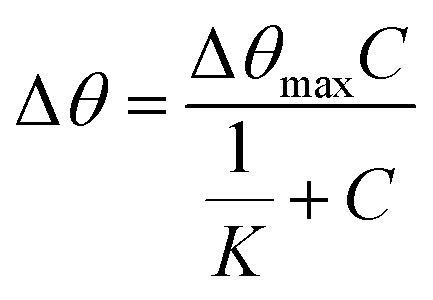
where *C* is the Zn^2+^ concentration, Δ*θ*_max_ is the maximum SPR shift at saturation, and *K* is the binding affinity constant. Fig. 14 shows the plot that fitted the Langmuir model for both modified and unmodified gold thin films. The Δ*θ*_max_ of the curve fitting SPR for the modified-gold thin film was 0.2617°, slightly higher than the maximum angle of experimental value, *i.e.*, 0.2536° with an *R*^2^ of 0.95.

The binding affinity was from the Langmuir model for Zn^2+^ towards the modified-gold thin film also was calculated and the value of *K* obtained was 1.927 × 10^3^ M^−1^, while for Zn^2+^ towards the unmodified gold thin film was 0.99 M^−1^.^62,63^ The higher affinity of Zn^2+^ ions towards the modified gold thin film proved that the modification of the gold thin with the NCC-based material helps to improve the sensitivity of the SPR optical sensor.

### 3.5. Affinity comparison of Zn^2+^ with other metal ions

**Fig. 15:**
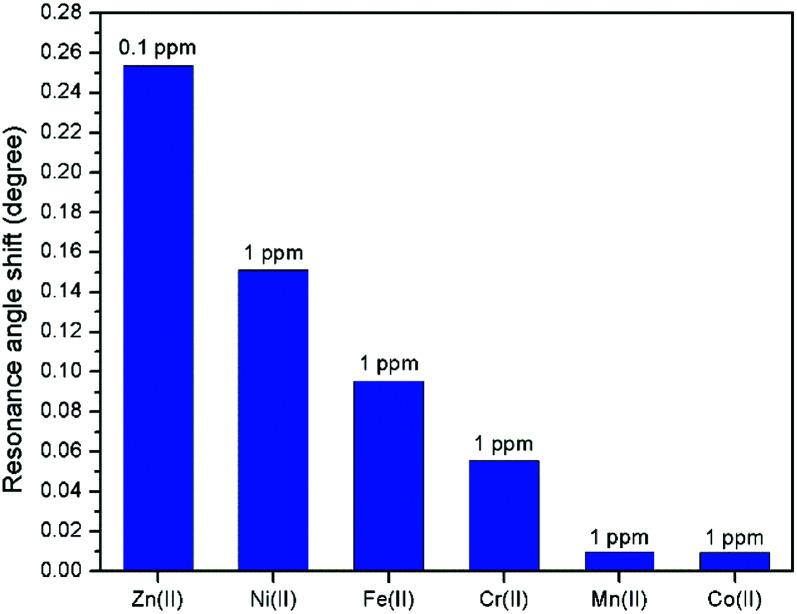
Affinity comparison of Zn^2+^ with other metal ions.

Further investigation was performed to study the affinity of other metal ions towards the modified-gold thin film for comparison with Zn^2+^. Fig. 15 depicts the shift in the resonance angle comparison of Zn^2+^ with different metal ions includes Ni^2+^, Fe^2+^, Cr^2+^, Mn^2+^, and Co^2+^. The concentration of Zn^2+^ was set at a lower concentration, *i.e.*, at 0.1 ppm while maintaining other metal ions concentration at 1 ppm. From Fig. 15, Zn^2+^ has the highest shift in the resonance angle at 0.2536° even at the concentration that is 10 times lower as compared to other metal ions at a higher concentration, which were 0.151°, 0.0955°, 0.0555°, 0.0097°, and 0.0096° for Ni^2+^, Fe^2+^, Cr^2+^, Mn^2+^, and Co^2+^, respectively. These results show that the SPR has a higher affinity towards Zn^2+^ using the modified-gold thin film.

## 4. Conclusions

In this study, the sensitivity of the SPR optical sensor was successfully enhanced by modifying the gold thin film with a nanocrystalline cellulose (NCC)-based material for Zn^2+^ detection. When the unmodified gold thin film was tested with the SPR in the first part of the experiment, the resonance angle remained the same at 53.66° with all concentrations of Zn^2+^. The second part of the SPR experiment proved that the modification of the gold thin film enhanced the sensitivity of the SPR, where the optical sensor was able to detect Zn^2+^ as low as 0.01 ppm. Also, the potential interactions of the Zn^2+^ with the modified-gold thin film also were studied *via* X-ray photoelectron spectroscopy. From the XPS results, it was determined that the Zn^2+^ ions may have interacted with the negative charge functional groups contained on the modified thin film surface, *i.e.*, COO^−^ and SO_3_^−^, *via* electrostatic interactions. Moreover, the sensitivity of the SPR using the modified-gold thin film was also calculated by comparing the resonance angle shift with concentrations of Zn^2+^. The SPR using the modified-gold thin film had a sensitivity of 1.892° ppm^−1^ with the lowest detection of 0.01 ppm compared to other previous studies. Besides that, the modified-gold thin film has a higher binding affinity compared to the unmodified gold thin film when calculated using the Langmuir isotherm model, *i.e.*, 1.927 × 10^3^ M^−1^ and 0.99 M^−1^, respectively. By using the modified-gold thin film, the SPR responses also had a higher affinity towards Zn^2+^ compare to Ni^2+^, Fe^2+^, Cr^2+^, Mn^2+^, and Co^2+^.

## Conflicts of interest

There are no conflicts of interest to declare.

## Supplementary Material
